# Prognostic nomogram and epidemiological analysis for lung atypical carcinoid: A SEER database and external validation study

**DOI:** 10.1002/cam4.6794

**Published:** 2023-12-20

**Authors:** Xinglin Yi, Yi He, Gangzhen Qian, Caixia Deng, Jiayi Qin, Xiangdong Zhou, Hu Luo

**Affiliations:** ^1^ Department of Respiratory Medicine Third Military Medical University Southwest Hospital Chongqing China; ^2^ Department of Cardiovascular Medicine Third Military Medical University Southwest Hospital Chongqing China; ^3^ Department of Gastroenterological Medicine Third Military Medical University Southwest Hospital Chongqing China

**Keywords:** atypical pulmonary carcinoids, epidemiology, nomogram, prognostic factor, SEER database

## Abstract

**Purpose:**

Our study aims to delineate the epidemiological distribution of pulmonary carcinoids, including atypical carcinoid (AC) and typical carcinoid (TC), identify independent prognostic factors, develop an integrative nomogram and examine the effects of various surgical modalities on atypical carcinoid‐specific survival (ACSS).

**Methods:**

Joinpoint regression model and age‐group distribution diagram were applied to determine the epidemiological trend of the pulmonary carcinoids. Univariate and least absolute shrinkage and selection operator (LASSO)‐based Cox regression models were used to identify independent factors, and a nomogram and web‐based predictor were developed to evaluate prognosis of AC patients individually. We performed Kaplan–Meier survival analyses to compare the scope of various surgical interventions, with and without G‐computation adjustment, utilising restricted mean survival time (RMST) to assess survival disparities.

**Results:**

A total of 1132 patients were recruited from the Surveillance, Epidemiology, and End Results database (SEER) and a separate medical centre in China. The mean age of AC patients was 63.4 years and a smoking history was identified in 79.8% of AC patients. Joinpoint analysis shows rising annual rates of new AC and carcinoid cases among lung cancers. Both the proportion of pulmonary TC and AC within the total lung cancer population exhibits an L‐shaped trend across successive age groups. The nomogram predicted 1, 3 and 5 years of AC with excellent accuracy and discrimination. Kaplan–Meier survival analyses, conducted both pre‐ and post‐adjustment, demonstrated that sublobar resection's survival outcomes were not inferior to those of lobectomy in patients with stage I‐II and stage III disease.

**Conclusion:**

This study is the first to reveal epidemiological trends in pulmonary carcinoids over the past decade and across various age cohorts. For patients with early‐stage AC, sublobar resection may be a viable surgical recommendation. The established nomogram and web‐based calculator demonstrated decent accuracy and practicality.

## 1 INTRODUCTION

Pulmonary atypical carcinoid (AC) is a rare and malignant type of neuroendocrine tumour that develops in the lungs and represents approximately 10%–15% of carcinoid neoplasms of the lung.^1^, ^2^ In 1998, Travis et al. defined AC that necessitates mitotic counts with necrosis from 2 to 10 per 2 mm^2^ of viable tumour, whereas typical carcinoids (TC) are <2 per 2 mm^2^.^3^ This increased activity can result in faster tumour growth in AC, usually considered as an intermediate‐grade tumour with a higher risk of spreading, a higher rate of recurrence and worse survival than TC, which is regarded as low‐grade tumour.^4^, ^5^

AC is typically diagnosed between the ages of 58 and 65 years and is more common in women than in men.^2^, ^6^ Symptoms of atypical pulmonary carcinoid include cough, chest pain, shortness of breath, fatigue and recurrent respiratory infections. Previous studies have indicated that AC patients frequently manifest symptoms indicating an incipient stage of the disease and that surgical excision is the preferred therapeutic approach.^7^ Nonetheless, the existing literature on AC largely emanates from surgical samples, liable to engender a partiality toward earlier‐stage disease and, consequently, more favourable prognoses. Because of the disease's rarity and the efficacy of radiation therapy and chemotherapy, whether administered in isolation or as an adjunct to surgical intervention, AC remains uncertain.^8^

Although the tumour node metastasis (TNM) system is now widely employed to forecast lung cancer survival, its predictive efficacy in patients with AC remains unverified because of the rarity of AC. In addition, risk factors, such as age, operation scheme and primary tumour site, identified in previous studies, can affect the prognosis of AC, suggesting that predictions relying on only one indicator may be inaccurate. Of note, the comparative impact of diverse surgical modalities on the survival of patients with AC is yet to be conclusively established, partly due to the relative rarity of these tumours. Recent studies found that sublobar resection, including segmentectomy and wedge resection, was not inferior to lobectomy for AC‐related survival.^9^, ^10^ Nonetheless, this conclusion may be arbitrary due to limited sample size in certain surgical approach and absence of adjustment methodologies to account for confounders within their Kaplan–Meier survival analyses. Therefore, a more definitive survival analysis could be achieved with an expanded cohort and refined analytical techniques.

Our study had three objectives. The first was to investigate the epidemiological distribution of pulmonary carcinoids, including AC and TC, to better understand the epidemiology of pulmonary carcinoids. This is pivotal for the early identification of the disease and comprehension of its aetiology. The second objective was to ascertain the independent prognostic factors for AC and to develop a comprehensive nomogram that could reliably predict patient prognosis. The third objective was to evaluate the influence of various surgical approaches across different TNM stages on the survival of patients with AC, utilising an appropriate strategy. Data from a multicentre database were employed to construct and validate this intricate and precise model.

## 2 METHODS

### 2.1 Patients and data collection

We conducted a retrospective study to collect enough patient information, using data from the Surveillance, Epidemiology, and End Results (SEER) database, including information on approximately 30% of cancer patients in the United States.^11^ The clinicopathological and long‐term follow‐up data in this database are extensive. Additionally, we included patients with AC from Southwest Hospital, which admit tens of thousands of individuals annually. Two distinct patient enrolment criteria were incorporated based on the objectives of this study. All patients diagnosed with AC or TC were included in the International Classification of Diseases for Oncology, 3rd edition (ICD‐O‐3) codes 8249 and 8240 for the epidemiological analysis, regardless of any missing clinicopathological data. Regarding the survival analysis, the inclusion criterion for our study was ICD‐O‐3 code 8249. The exclusion criteria included patients with unknown information on tumour size, TNM stage, primary tumour site, laterality and metastatic sites and those lacking data on age, surgical status and survival time. Subsequently, a comprehensive epidemiological analysis was conducted using data from the SEER database, including 1367 AC patients and 11,951 TC patients. Additionally, survival analysis was performed on a separate dataset consisting of 1008 patients from the SEER program and 124 patients from Southwest Hospital. (Figure S1).

Several variables, including age, gender (male/female), laterality (left/right), primary tumour site (lower/main bronchus/middle/upper/overlapping lesion), marital status (married/ other), T stage (T1‐4), N stage (N0‐3), M stage (M0‐1), surgery (lobectomy/no/ pneumonectomy/sublobar resection), chemotherapy (yes/no/unknown) and radiation (yes/no/unknown) were collected for survival analysis. In our study, we used the 8th edition of the TNM staging system to uniformly stage tumours. We used AC‐specific survival (ACSS), defined as the period from the diagnosis of AC to the death specifically caused by AC, to describe patient survival with AC.

### 2.2 Epidemiological patterns investigation

In this part, we conducted joinpoint regression analysis to investigate the percentage trend of pulmonary carcinoids, including AC and TC. Apart from joinpoint analysis, the age‐group distribution diagram of TC and AC was also performed with aim to observe the TC and AC proportion trends in the total lung cancer population. In the joinpoint analysis, the annual percentage change (APC) and average APC (AAPC) were determined using linear regression, with each year serving as an independent variable. Using a weighted APC, AAPC serves as a comprehensive measure of trends across a predefined time interval, enabling us to succinctly summarise the AAPCs over several years using a single figure. The AAPC value indicates whether there has been an increase, decrease or no change and the annual percentage change. For instance, an AAPC of 5 suggests a 5% annual rate of increase. A joinpoint refers to the specific point at which a trend is segmented into multiple parts, each representing a distinct phase of change in the trend. The JoinPoints regression program software was used to determine the optimal number of joinpoints. The statistical significance of the JoinPoints regression analysis was confirmed if the *p*‐value was <0.05.

### 2.3 Identification of independent factors

Before conducting the analysis, we divided all individuals enrolled from the SEER database into two groups in a 7:3 ratio and randomly assigned them to either the training or internal validation cohort. We also designated individuals from the Southwest Hospital as the external validation cohort. The independent variables were identified exclusively within the training cohort. To reduce overfitting, we conducted univariate Cox regression to identify possible correlating variables by incorporating these features to determine the independent variables and the corresponding hazard ratio (HR) with ACSS, followed by least absolute shrinkage and selection operator (LASSO) Cox regression with 10‐fold cross‐validation to stabilise it. In this analysis, the penalty factor (lambda) is crucial in regulating the extent of shrinkage applied to the regression coefficients of the predictor variables. The average test error is then computed for each value of lambda, and the value that yields the lowest error is chosen as the optimal lambda, where several coefficients of multiple variables remain non‐zero, signifying their importance.

### 2.4 Nomogram development and validation

A nomogram evaluating 1‐, 3‐ and 5‐year survival probability was established by incorporating independent variables into Cox regression. Clinicians can use the nomogram by vertically drawing a line extending from each independent variable up to the upper axis labelled ‘points’, which provides the corresponding points for each variable. The total score was calculated by summing the scores for each independent variable. To determine the individual survival probability at 1, 3 and 5 years, clinicians draw a vertical line from the ‘total score’ axis to the survival probability line. The column located above each variable line and ‘Total score’ displays the population distribution.

The assessment procedure was performed in the internal and external validation arms and included indices such as the receiver operating characteristic curve (ROC), the area under the ROC (AUC), calibration plots and decision curve analysis (DCA). ROC and AUC are indicators of accuracy evaluation; the closer they are to 1, the higher the accuracy of the model, whereas the closer they are to 0, the worse the prediction accuracy of the model. Calibration is the degree to which the model's predicted probabilities accurately reflect the true probabilities of events of interest. It is useful for identifying whether a model is well‐calibrated or tends to overpredict or underpredict probabilities. The closer the points on the plot are to the diagonal line, the better the model's calibration. DCA is a statistical method used to evaluate and compare predictive models and provides a way to quantify the clinical value by estimating the net benefit of its use across a range of possible decision thresholds. These analyses were performed both for the nomogram and TNM stage because this can help us understand how TNM staging behaves for patients with AC and show the accuracy of the nomogram. Finally, we developed and published a web‐based calculator that used a nomogram. Anyone visiting can easily and without payment access a nomogram to evaluate an individual's survival with AC.

### 2.5 Kaplan–Meier survival analysis of various surgical approaches and restricted mean survival time (RMST)

In order to investigate the survival disparities among various surgical approaches, we performed the Kaplan–Meier survival analyses with and without the G‐computation method or direct adjustment.^12^, ^13^ Here, the direct adjusted probabilities could estimate likelihoods of outcomes in populations with similar prognostic or predictive co‐variates. These adjustments are important when we tried to imply causal inference which we discussed in a prior typescript in this series.^14^, ^15^ After Kaplan–Meier analyses of different treatments, we used RMST to evaluate the effect of an intervention over the entire duration of follow‐up. Here, RMST is a sophisticated statistical measure used in the analysis of survival data, summarising the entire area under the survival curve up to a specified time point. It could provide an intuitive, clinically relevant measure of average expected survival time without meeting standard of proportional hazards assumption.^16^, ^17^

### 2.6 Statistical analysis

The analysis in this study was completed using R software (v. 4.2.1) and the Joinpoint Regression Program (v. 4.9.1). The uni‐ and multivariate Cox analysis is performed under packages including ‘survival’, ‘glmnet’, ‘broom’ and ‘forplo’; The model establishment and assessment processes are undergone under packages including ‘regplot’, ‘DynNom’, ‘rms’, ‘survival’, ‘survminer’, ‘adjustedCurves’ and ‘pec’. In the term of development of web‐based calculator, we first saved and input the training data into a R script, then ‘DynNom’ package in R software was utilised to set as a background layout design, where we set a list of buttons with the same name of variables in the training data. Then we write in the same script to establish a Kaplan–Meier using the ‘survival’ and ‘survminer’ packages and to develop a corresponding barplot using ‘ggplot2’ package. Finally, this script was released online through the ‘shinyapp’, which is an open‐source R package that provides an elegant and powerful web framework for building web applications using R. The detailed script was available through the Supplementary materials.

## 3 RESULTS

### 3.1 Epidemiological trend of AC

**FIGURE 1:**
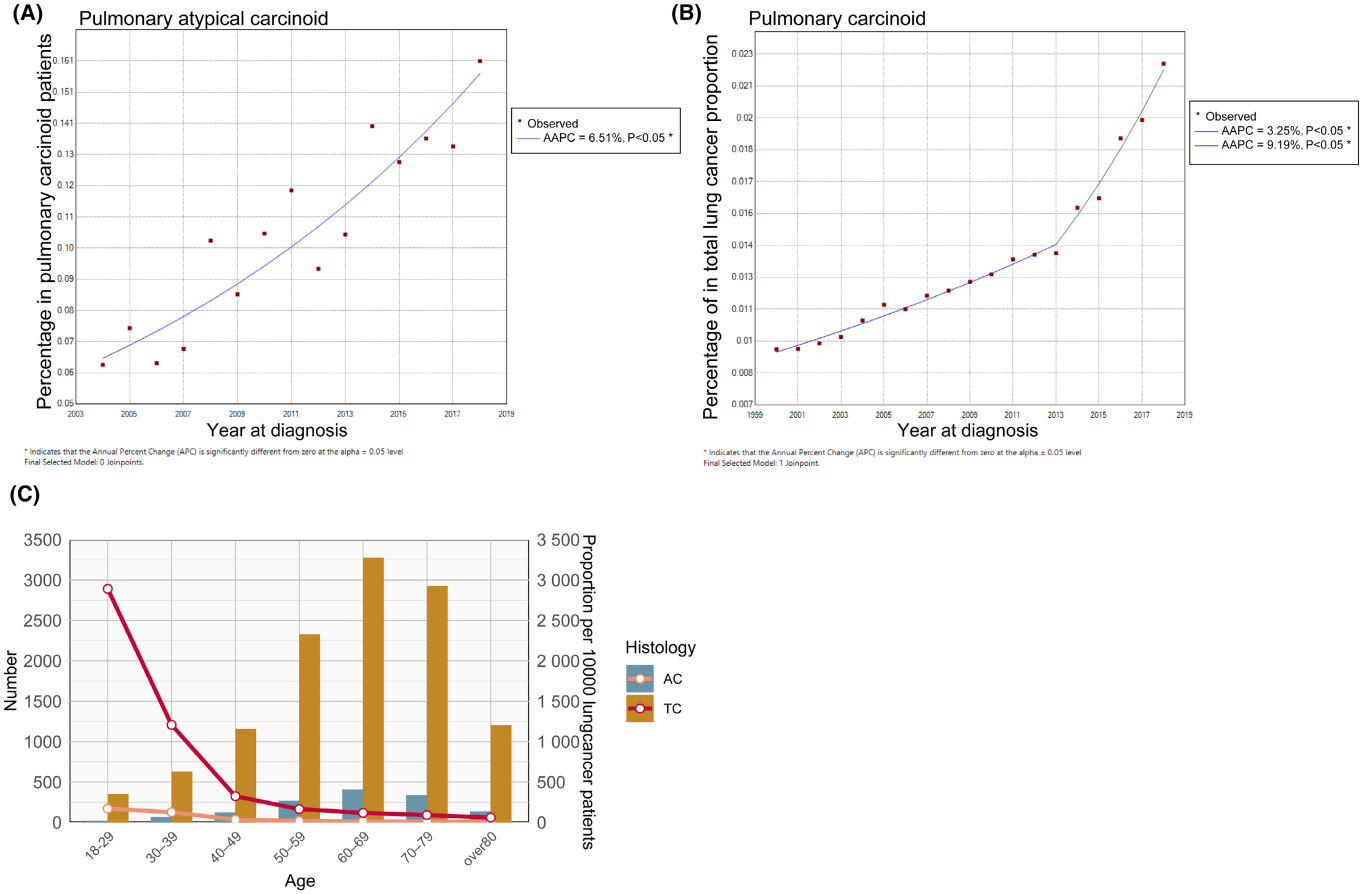
Epidemiological patterns of pulmonary carcinoids. Joinpoint regression analysis of the annual percentage trend of newly diagnosed AC in total pulmonary carcinoid cases (A); Joinpoint regression analysis of the annual percentage trend of newly diagnosed pulmonary carcinoids in total lung cancer cases (B); age‐group distribution of AC numbers and their proportion in total lung cancer cases (C). AC, atypical carcinoid; AAPC, average annual percentage change.

Annually, the proportion of patients with newly diagnosed atypical carcinoid (AC) among pulmonary carcinoid cases rose from 6.51% in 2004 to 10.5% in 2010, reaching 16.1% in 2018. The optimal number of join points was zero, indicating that there was only one trend of percentage change. Figure 1A showed that the percentage of AC in lung carcinoid increased gradually between 2004 and 2018 with an AAPC value of 6.5% (95% CI: 4.9%–8.2%, *p* < 0.001). Figure 1B indicates that, within the total new lung cancer cases, the percentage of pulmonary carcinoids cases progressively rose from 0.95% in 2000 to 1.37% in 2013, exhibiting an AAPC of 3.25%. Post‐2013, this uptrend accelerated, reaching 2.23% by 2018, with an AAPC of 9.19%. Both periods of increase were statistically significant, with *p‐*values <0.05. Figure 1C illustrates that the predominant age range for diagnosis of both TC and AC tumours is 50–79 years, with a particular concentration in the 60–69 age group. Contrary to expectations, an analysis of the age‐distribution proportion of pulmonary carcinoids tumours within the total lung cancer cohort reveals an L‐shaped trend across successive age brackets. Specifically, for individuals aged 18–29, the incidence of TC and AC was 2892 and 172 per 10,000 lung cancer cases, respectively. These figures declined to 1208 for TC and 127 for AC among the 30–39 age group and further to 324 and 347 in the 40–49 age group. Notably, despite the mean age at diagnosis being approximately 60–69 years, the proportions of TC and AC within this demographic were comparatively low, at 118 and 14.7 per 10,000 lung cancer cases, respectively.

### 3.2 Baseline for patients with AC for survival analysis

Following the screening procedure, 1132 patients with AC were enrolled in the survival analysis. As presented in Table 1, most AC patients were women (60.5%) and white (78.4%), with tumours located on the right side (58.7%) and in the lower region of the lung (42.7%). The patients were diagnosed at an average age of 63.4 years, with early T (51.5%), *N* (64.4%) and M (87.1%) stages. Lobectomy was the most common surgical procedure (57.2%), and only a few patients underwent chemotherapy (20.9%) or radiotherapy (13.0%). Most variables did not differ significantly between the SEER and Southwest Hospital cohorts, except for sex, M stage, race, marital status, age and surgery. An overview of the distribution of characteristics is displayed as a heat map (Figure S2). The cohort analysis from Southwest Hospital showed a high prevalence of smoking, with 79.8% of patients being current smokers or having a history of smoking. However, our statistical evaluation found no significant correlation between smoking status and various clinical stages: There was no significant association with T stage (*p* = 0.458), N stage (*p* = 0.940), M stage (*p* = 0.848), or ACSS (*p* = 0.267), as detailed in Table S1.

**TABLE 1:** Baseline characteristics of patients with AC.

Characteristics	Overall (*N* = 1132)	SEER cohort (*N* = 1008)	External validation cohort (*N* = 124)	*p*‐value
Sex				
Female	685 (60.5%)	665 (66%)	40 (32.3%)	**<0.001**
Male	447 (39.5%)	343 (34%)	84 (67.7%)	
Laterality				
Left	468 (41.3%)	407 (40.4%)	61 (49.2%)	0.074
Right	664 (58.7%)	601 (59.6%)	63 (50.8%)	
T stage				
T1	583 (51.5%)	522 (51.8%)	61 (49.2%)	0.89
T2	293 (25.9%)	258 (25.6%)	35 (28.2%)	
T3	156 (13.8%)	140 (13.9%)	16 (12.9%)	
T4	100 (8.8%)	88 (8.7%)	12 (9.7%)	
N stage				
N0	729 (64.4%)	649 (64.4%)	80 (64.5%)	0.47
N1	162 (14.3%)	143 (14.2%)	19 (15.3%)	
N2	215 (19.0%)	195 (19.3%)	20 (16.1%)	
N3	26 (2.3%)	21 (2.1%)	5 (4%)	
M stage				
M0	986 (87.1%)	886 (87.9%)	100 (80.6%)	**0.033**
M1	146 (12.9%)	122 (12.1%)	24 (19.4%)	
Primary site				
Lower	483 (42.7%)	433 (43%)	50 (40.3%)	0.802
Main bronchus	48 (4.2%)	44 (4.4%)	4 (3.2%)	
Middle	196 (17.3%)	173 (17.2%)	23 (18.5%)	
Overlapping lesion	19 (1.7%)	18 (1.8%)	1 (0.8%)	
Upper	386 (34.1%)	340 (33.7%)	46 (37.1%)	
Race				
Black	89 (7.9%)	89 (8.8%)	0 (0%)	**<0.001**
Other	156 (13.8%)	32 (3.2%)	124 (100%)	
White	887 (78.4%)	887 (88%)	0 (0%)	
Age (Mean ± SD)	63.4 ± 12.7	63.2 ± 12.9	65.5 ± 10.1	**0.017**
Marital status				
Married	688 (60.8%)	564 (56%)	124 (100%)	**<0.001**
Other	444 (39.2%)	444 (44%)	0 (0%)	
Surgery				**<0.001**
Lobectomy	648 (57.2%)	585 (58%)	63 (50.8%)	
No	244 (21.6%)	188 (18.7%)	56 (45.2%)	
Pneumonectomy	38 (3.4%)	36 (3.6%)	2 (1.6%)	
Sublobar resection	202 (17.8%)	199 (19.7%)	3 (2.4%)	
Chemotherapy				0.126
No/Unknown	895 (79.1%)	804 (79.8%)	91 (73.4%)	
Yes	237 (20.9%)	204 (20.2%)	33 (26.6%)	
Radiation				0.865
No/Unknown	985 (87.0%)	876 (86.9%)	109 (87.9%)	
Yes	147 (13.0%)	132 (13.1%)	15 (12.1%)	
Smoking				
No			25 (20.2%)	
Yes			99 (79.8%)	

*Note*: The bold values indicate *p*‐value is less than 0.05.

### 3.3 Identification of the independent features of ACSS

**FIGURE 2:**
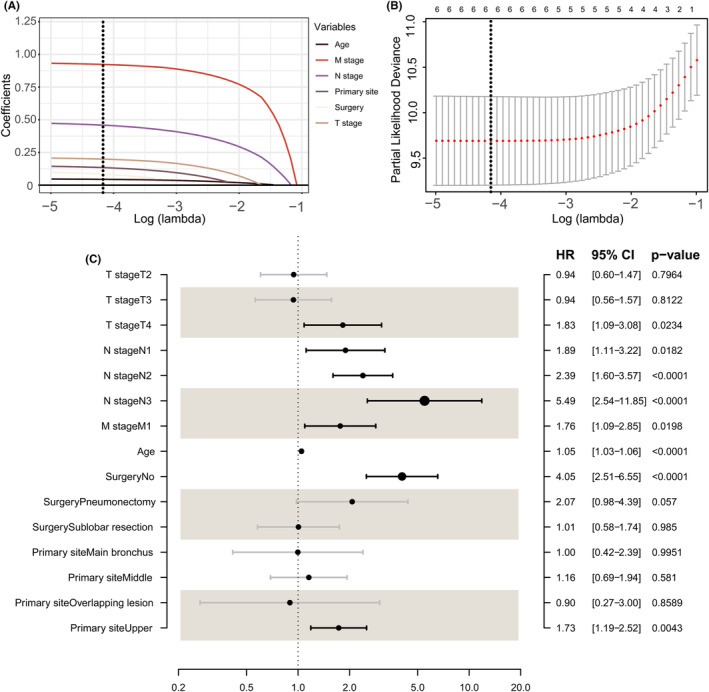
LASSO Cox regression. Plot of coefficients for multiple variables (A). Selection of optimal lambda with 10‐fold cross‐validation (B). Multivariate Cox forest plot (C) LASSO, least absolute shrink and selection operator.

Univariate Cox regression forest plot (Figure S3) showed that age (HR: 1.05, *p* < 0.001), T4 (HR: 1.76, *p* = 0.036), N1 (HR: 1.82, *p* = 0.031), N2 (HR: 2.32, *p* < 0.001), N3 (HR: 4.79, *p* < 0.001), M1 (HR: 1.77, *p* = 0.021), upper tumour site (HR: 1.79, *p* = 0.003), no surgery (HR: 4.03, *p* < 0.001) and pneumonectomy (HR: 2.24, *p* = 0.038) are parameters contributing significantly to ACSS. In the LASSO‐based multivariate Cox regression, we found that as the optimal log (lambda) was reached (Figure 2A,B), six variables remained non‐zero, which were then considered independent, including age (HR: 1.05, *p* < 0.001), T4 (HR: 1.83, *p* = 0.023), N1 (HR: 1.89, *p* = 0.018), N2 (HR: 2.39, *p* < 0.001), N3 (HR: 5.49, *p* < 0.001), M1 (1.76, *p* = 0.020), upper tumour site (HR: 1.73, *p* = 0.004) and no surgery (HR: 4.05, *p* < 0.001) (Figure 2C).

### 3.4 Nomogram development and assessment

**FIGURE 3:**
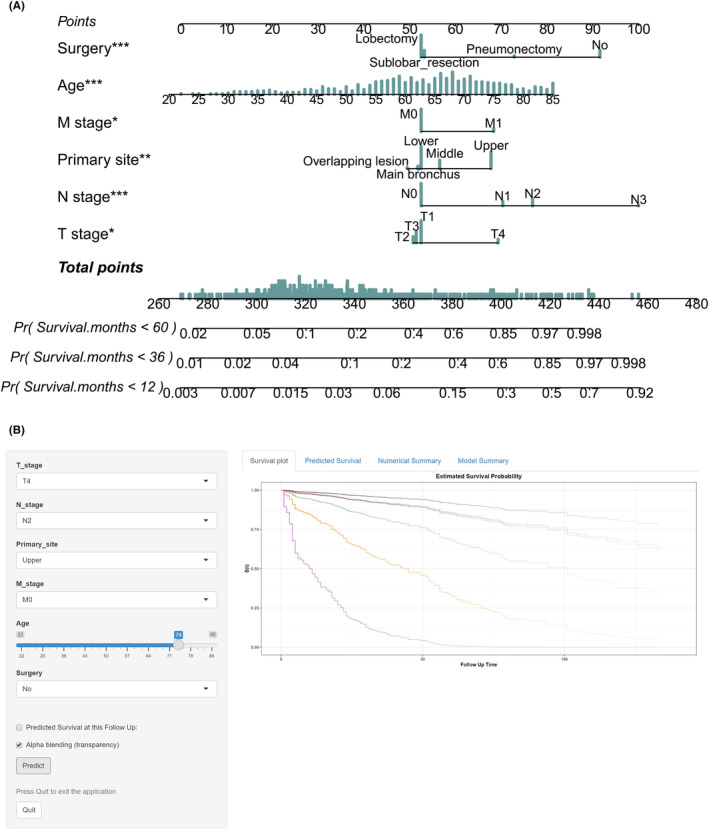
Nomograms for ACSS. A nomogram for predicting the survival probability at 1, 3 and 5 years of AC patients (A) and a web‐based nomogram predicting the survival curve of AC patients (B).

After selecting independent variables through univariate and LASSO Cox regression analyses, we successfully created a nomogram that could predict ACSS at 1, 3 and 5 years (Figure 3A). Moreover, we developed a web‐based calculator using the nomogram, accessible through the website: https://machinelearningxly.shinyapps.io/PSCC/. As illustrated in Figure 3B, survival curves were promptly obtained for each patient's prognosis by simply choosing the relevant parameters from the dropdown menu.

**FIGURE 4:**
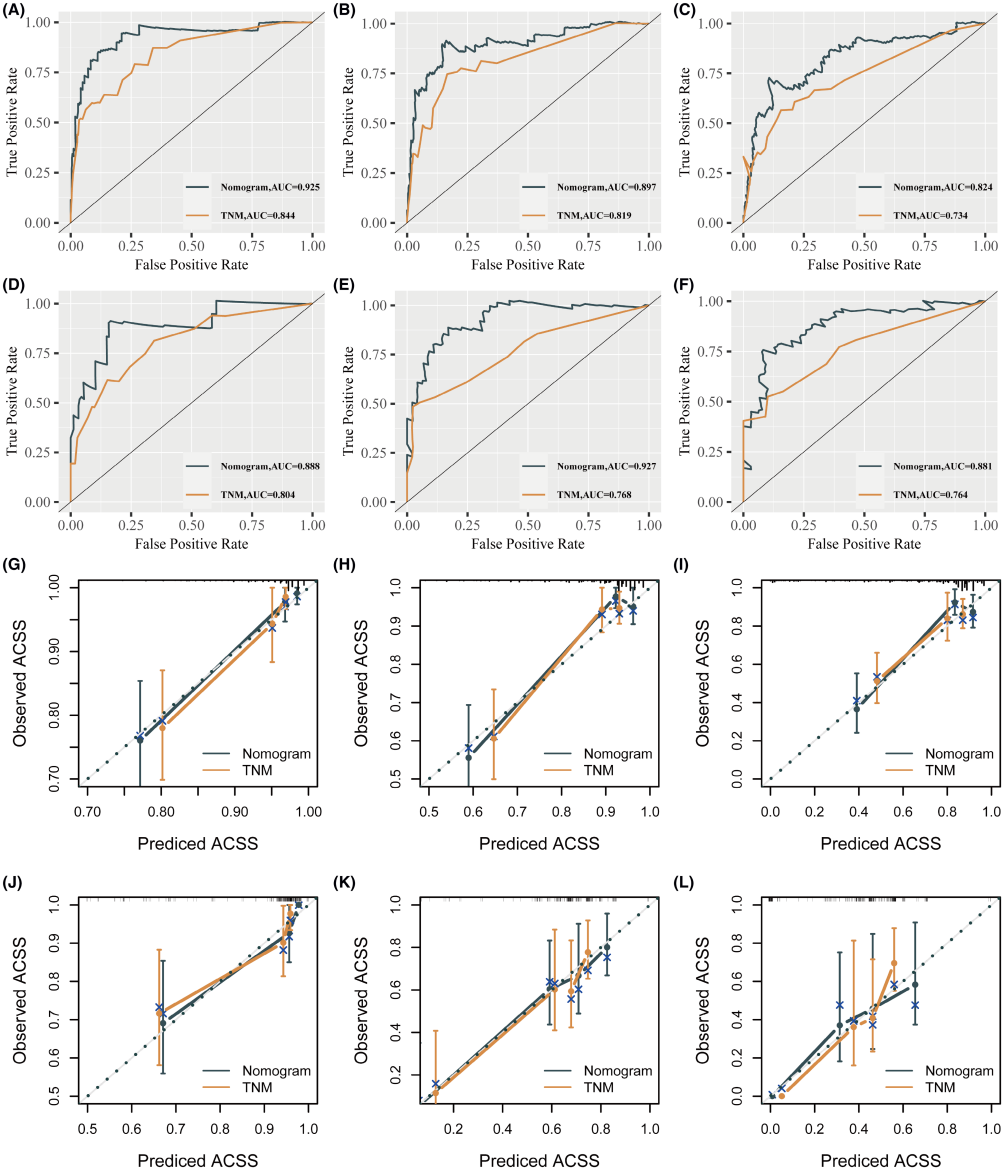
Evaluation of nomogram. ROCs of nomogram and TNM stage at 1, 3 and 5 years in the internal validation arm (A–C) and in the external validation arm (D–F). Calibration plots of nomogram and TNM stage at 1, 3 and 5 years in the internal validation arm (G–I) and in the external validation arm (J–L).

The results depicted in Figure 4 demonstrate that the nomogram's AUC achieved scores in the internal validation arm, with values of 0.925 (Figure 4A), 0.897 (Figure 4B) and 0.824 (Figure 4C) at 1, 3 and 5 years, respectively, while it yielded AUC values of 0.888 (Figure 4D), 0.927 (Figure 4E) and 0.881 (Figure 4F) in the external validation arm. These outcomes seemed to significantly exceed those of the TNM stage, with AUC values of 0.844 (Figure 4A), 0.819 (Figure 4B) and 0.734 (Figure 4C) in the internal validation arm and 0.804 (Figure 4D), 0.768 (Figure 4E) and 0.764 (Figure 4F) in the external validation arm. Calibration plots demonstrated that the predicted results of the nomogram were consistent with the actual outcomes in the internal (Figure 4G–I) and external (Figure 4J–L) validation cohorts. The DCA curve indicates that the nomogram has high clinical utility, and the area under the DCA curves seems significantly higher than that of the TNM stage, regardless of the internal (Figure S4A–C) and external validation arms (Figure S4D–F).

### 3.5 Kaplan–Meier survival analysis of various surgical approaches and restricted mean survival time (RMST)

**FIGURE 5:**
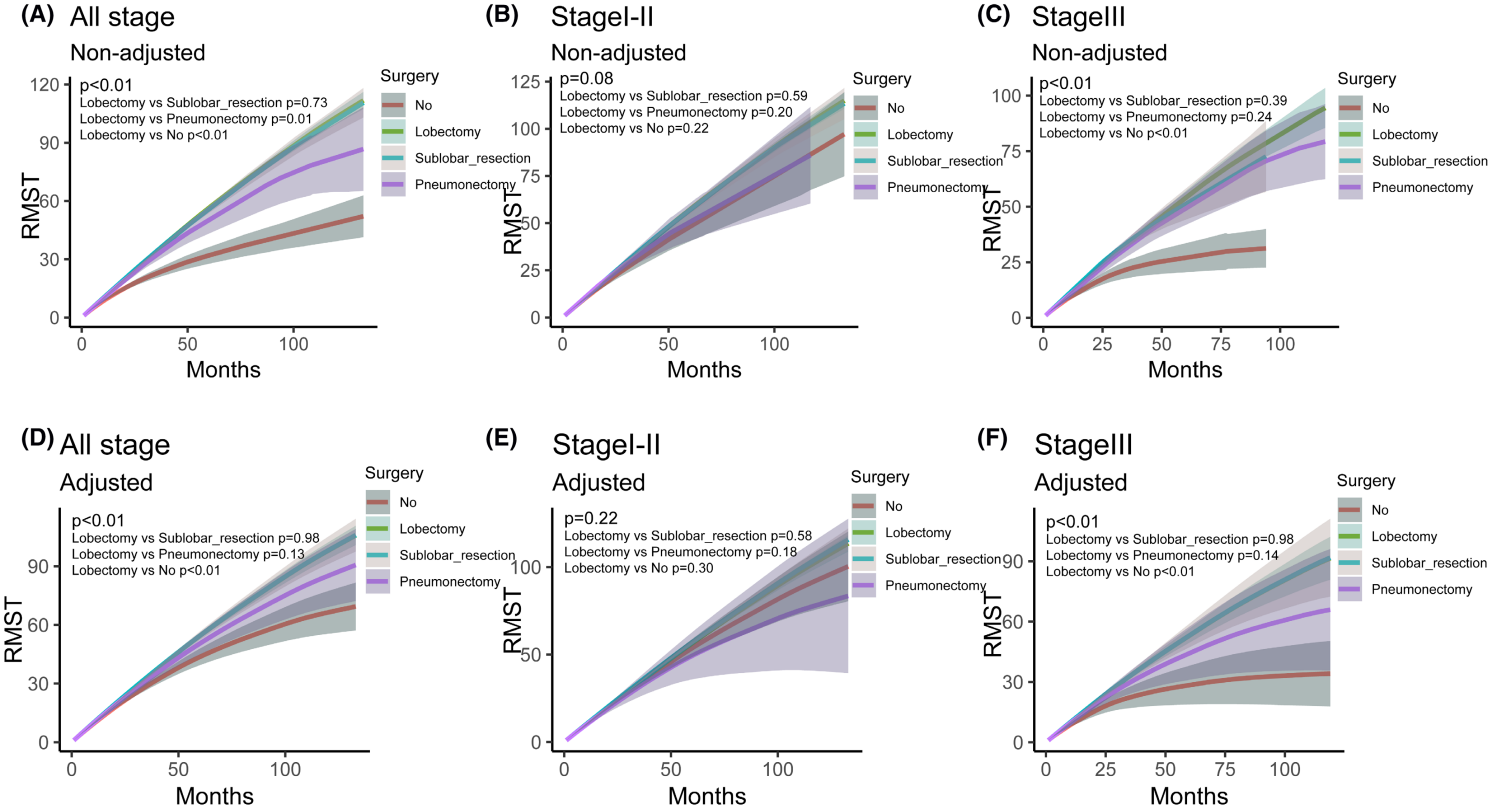
Discrepancies in RMST across surgical approaches. The RMST comparisons in all stage (A), stage I‐II (B) and stage III (C) in the adjusted Kaplan–Meier analysis based on G‐computation method; the RMST comparisons in all stage (D), stage I‐II (E) and stage III (F) in the unadjusted Kaplan–Meier analysis. RMST, restricted mean survival time.

Figure S5 presents the Kaplan–Meier curves for patient survival both before and after adjustment via G‐computation, with RMST outcomes depicted in Figure 5. Across all stages, stage I‐II and stage III of AC patients, pre‐adjustment analysis indicated that lobectomy and sublobar resection had similar survival trends (Figure 5A–C and Figure S5A,C,E). This similarity persisted even after adjustment (Figure 5D–F and Figure S5B,D,F).

In stage I‐II AC patients, no statistically significant differences in RMST were observed among the various surgical strategies, either before or after adjustment. However, there was a trend suggesting that both lobectomy and sublobar resection may offer more benefit to AC patients than either pneumonectomy or no surgery at all (Figure 5B,E and Figure S5C,D). In the cohort of stage III AC patients, a statistically significant variation was observed among different surgical treatments (*p* < 0.01). The comparison of RMST indicated that both lobectomy and sublobar resection are associated with improved outcomes relative to pneumonectomy; however, this improvement did not attain statistical significance. Nonetheless, when compared to non‐surgical management, both lobectomy and sublobar resection demonstrated a statistically significant enhancement in RMST. (Figure 5C,F and Figure S5E,F).

## 4 DISCUSSION

AC is a rare type of lung cancer, and its clinical characteristics and underlying molecular mechanisms are poorly understood. The key distinguishing feature between AC and TC tumours is an increase in tumour mitosis and necrosis.^18^ Utilising joinpoint regression analysis, this study has documented a steady increase over the past two decades in the percentage of newly diagnosed AC cases among newly identified pulmonary carcinoids tumours, with an AAPC value of 6.51%. Additionally, the percentage of newly diagnosed pulmonary carcinoids in relation to total new lung cancer cases has also exhibited a significant increase during the same period, escalating from 0.95% to 2.23%. This increase became more pronounced after 2013. These increasing trends may be indicative of heightened clinical awareness and improvements in the pathological identification of this relatively rare subtype. It may also signal a worrisome correlation with heightened environmental exposures over recent decades, such as augmented ambient particulate matter pollution and a rise in the prevalence of high body mass index.^19^ This pattern underscores the necessity for further research into the pathogenesis and aetiology of pulmonary carcinoids.

Interestingly, our data demonstrate that the representation of both TC and AC within the total lung cancer demographic adheres to an L‐shaped distribution across successive age groups. Our findings suggest a decreasing trend in the representation of pulmonary carcinoids tumours with advancing age. The proportion of lung cancer diagnoses attributable to AC is nearly one third in the 18–29 age group, decreasing to one eighth in the 30–39 age group and further to 3.6% in the 40–49 age group. The observed pattern reveals that, while the average age of diagnosis for AC tumours falls within the 60–69 age group, this age group comprises only 1.3% of all lung cancer cases. This indicates that AC tumours are not as prevalent in the expected age demographic as they are in younger age groups. The reasons behind this age‐related distribution are not immediately clear, emphasising the need for more comprehensive studies to elucidate the underlying mechanisms.

Although the TNM stage showed good discriminative ability for ACSS, a nomogram developed in current study by adding other independent variables and found an excellent result, displaying a much higher accuracy and clinical application value than the TNM stage. Furthermore, the web predictor was another achievement of this study that helped accurately capture the prognosis of patients with AC.

Previous studies have suggested that patients with AC are more likely to be older, smoker and have lymphatic metastasis than those with TC.^20^, ^21^ In this study, a higher prevalence of smoking history was noted among AC patients, yet no statistical significance observed when correlating smoking with TNM stage. Additionally, the majority of AC cases were women, with tumours predominantly located on the right side and lower region of the lung, corroborating the findings of Chen et al.^2^ This distribution of characteristics was similarly observed in patients with TC, as detailed in the analysis by Yoon et al.^6^ Universally, AC is associated with a poorer prognosis relative to TC, which may be partially attributed to its presentation at more advanced TNM stages. Dermawan et al. disclosed that out of 205 patients with pulmonary carcinoids, the 17 who had AC experienced shorter survival times and a significantly higher recurrence rate (8/17) within a 3‐year span, irrespective of TNM stage consideration.^22^ Furthermore, Yoon et al. reported that AC histology, accounting for 8% of lung carcinoid cases, had a higher proportion of late‐stage disease (stage III‐IV) at 65%, compared with 21% among TC patients. They posited that despite the absence of a statistically significant difference in the adjusted HR between stage II (HR: 2.6, 95% CI: 1.2–6.4) and stage III (HR: 3.7, 95% CI: 1.9%–8.4%), the eighth edition TNM staging could still provide a reliable prognostic indication for this rare lung cancer subtype, setting the stage for further research.^6^

Indeed, while TNM stage proved to be a significant factor, multivariate LASSO Cox regression analysis identified it was not the sole independent determinant of prognosis in AC patients. Other independent prognostic indicators included primary tumour site, age at diagnosis and surgical status. Our data support the significance of age as a prognostic marker for ACSS in AC patients. This is consistent with the findings of Wegner et al.,^23^ who, in their study of 662 patients with stage I‐III AC, observed an HR of 2.14 (*p* = 0.02) for ACSS. However, this contrasts with the findings in Wegner's study.^23^ In their Cox regression analysis of AC patients who underwent adjuvant therapy post‐surgery, age was not identified as a risk factor for ACSS. The discrepancy between these studies could be attributed to potential confounding factors related to older age that were not adjusted for, such as the presence of comorbidities, variations in tumour biology or differential responses to treatment. The lower lungs (42.7%) were the most common sites of AC, followed by the upper (34.1%) and middle (17.3%) lungs. In this study, the upper primary site of the AC, higher TNM stage and absence of surgery were also found to be adverse factors for ACSS, which has also been demonstrated in prior studies.^2^, ^23^ In addition, several studies have suggested that the mean mitotic count in a specimen can significantly affect the ACSS. Samples with 2–10 mitoses had a 5‐year recurrence‐free survival (RFS) rate of 94.6%, whereas samples with >10 mitoses had a rate of 75.2%.^24^ Similarly, according to Han et al., patients with fewer than five mitoses exhibited significantly longer median disease‐free survival (DFS) than those with more than five mitoses. Specifically, the former group had a median DFS of 52 months, whereas the latter had a median DFS of only 29 months.^25^

To evaluate the proper surgical approaches was also an important purpose of this study. The multivariate Cox regression demonstrated a worsening survival in patients without underwent surgery ([HR], 4.05; *p* < 0.001) compared with lobectomy treatment, but not in patients who underwent sublobar resection (*p* = 0.985). The RMST analysis of Kaplan–Meier survival curves indicated a similar survival trend between sublobar resection and lobectomy for patients in both early (stage I‐II) and advanced (stage III) stages, showing better outcomes compared to pneumonectomy or non‐surgical interventions. This aligns with the expert consensus advocating for complete anatomic resection (either sublobar resection or lobectomy) for non‐metastatic AC tumours.^26^ Support for this approach is further reinforced by another retrospective study employing propensity score matching, which demonstrated comparable long‐term survival between patients with early‐stage pulmonary carcinoids undergoing either sublobar resection or lobectomy.^27^ Concurring with Caplin's perspective,^26^ we acknowledge that the choice of surgery should be tailored to specific clinical conditions, such as increased mitotic counts or diminished pulmonary function. Moreover, despite the presence of liver metastasis, existing literature suggests that curative surgery is advisable for debulking if over 90% of the tumour can be excised, which can lead to a significant increase in 5‐year overall survival rates, potentially up to 70%.^28^

While this study is limited by the relatively small patient cohort for more detailed subgroup survival analysis, it boasts the largest patient sample to date, enhanced by the application of G‐computation adjustment. This provides valuable insights that complement the findings of Walters^9^ and Chen.^10^ Nonetheless, we recognise the intricacies of confounding factors and agree that further investigation is necessary to gain a deeper understanding of how surgical methods impact the prognosis of AC.

The study had few limitations. Although this study obtained relatively credible results based on a large population cohort, selection bias could not be avoided because of its retrospective nature. In addition, the specifics of key clinical variables that influence the prognosis of AC, including smoking habits and pathological results such as mitoses, remain unaccounted for. In the future, acquiring comprehensive data on these factors and extending the participants' population will be critical to ensure an accurate evaluation of the model.

In conclusion, our study identified a rising trend in the percentage of newly diagnosed patients with AC among the new pulmonary carcinoid patients, increasing from 6.51% in 2004 to 10.5% in 2010 and further to 16.1% in 2018, with an AAPC of 6.51%. Notably, the percentage of newly identified pulmonary carcinoids relative to total new lung cancer cases over the past two decades has also experienced a significant increase, escalating from 0.95% to 2.23%, with a more marked rise post‐2013. The age distribution diagram suggested the proportion of pulmonary TC and AC within the total lung cancer population exhibits an L‐shaped trend across successive age groups. Furthermore, sublobar resection, in comparison with lobectomy, did not demonstrate inferior survival for stage I‐II and stage III patients, both before and after G‐computation adjustment. Age at diagnosis, primary tumour site, surgical intervention and TNM stage were identified as independent risk factors for AC. The developed nomogram and web‐based predictor exhibited substantially greater accuracy and discriminative capability than the TNM staging alone.

## AUTHOR CONTRIBUTIONS

**Xinglin Yi:** Conceptualization (equal); data curation (equal); formal analysis (equal); investigation (equal); software (equal); supervision (equal). **Yi He:** Conceptualization (equal). **Gangzhen Qian:** Data curation (equal). **Caixia Deng:** Validation (equal). **Jiayi Qin:** Supervision (equal). **Xiangdong Zhou:** Supervision (equal). **Hu Luo:** Supervision (equal).

## FUNDING INFORMATION

This research was funded by the Chongqing Health Appropriate Technology Promotion Project (no. 2020jstg016).

## CONFLICT OF INTEREST STATEMENT

We admitted no conflict of interest.

## ETHICAL APPROVAL

The analysis was completed under approval of Southwest ethical board.

## Supporting information


Figure S1.

Figure S2.

Figure S3.

Figure S4.

Figure S5.

Table S1.

Data S1.

## Data Availability

Please contact the corresponding author with an appropriate reason.

## References

[1] He Y , Zhao F , Han Q , Zhou Y , Zhao S . Prognostic nomogram for predicting long‐term cancer‐specific survival in patients with lung carcinoid tumours. BMC Cancer. 2021;21(1):141. doi:10.1186/s12885-021-07832-6 33557782 PMC7871376

[2] Steuer CE , Behera M , Kim S , et al. Atypical carcinoid tumor of the lung: a surveillance, epidemiology, and end results database analysis. J Thorac Oncol. 2015;10(3):479‐485. doi:10.1097/JTO.0000000000000419 25371080

[3] Travis WD , Rush W , Flieder DB , et al. Survival analysis of 200 pulmonary neuroendocrine tumours with clarification of criteria for atypical carcinoid and its separation from typical carcinoid. Am J Surg Pathol. 1998;22(8):934‐944. doi:10.1097/00000478-199808000-00003 9706973

[4] Beasley MB , Thunnissen FB , Brambilla E , et al. Pulmonary atypical carcinoid: predictors of survival in 106 cases. Hum Pathol. 2000;31(10):1255‐1265. doi:10.1053/hupa.2000.19294 11070119

[5] Travis WD , Brambilla E , Burke AP , Marx A , Nicholson AG . Introduction to the 2015 World Health Organization classification of tumours of the lung, pleura, thymus, and heart. J Thorac Oncol. 2015;10(9):1240‐1242. doi:10.1097/JTO.0000000000000663 26291007

[6] Yoon JY , Sigel K , Martin J , et al. Evaluation of the prognostic significance of TNM staging guidelines in lung carcinoid tumours. J Thorac Oncol. 2019;14(2):184‐192. doi:10.1016/j.jtho.2018.10.166 30414942

[7] Kaifi JT , Kayser G , Ruf J , Passlick B . The diagnosis and treatment of bronchopulmonary carcinoids. Dtsch Arztebl Int. 2015;112(27–28):479‐485. doi:10.3238/arztebl.2015.0479 26214234 PMC4524963

[8] Bertino EM , Confer PD , Colonna JE , Ross P , Otterson GA . Pulmonary neuroendocrine/carcinoid tumours: a review article. Cancer. 2019;115(19):4434‐4441. doi:10.1002/cncr.24498 19562772

[9] Walters SL , Canavan ME , Salazar MC , et al. A National Study of surgically managed atypical pulmonary carcinoids Tumours. Ann Thorac Surg. 2021;112(3):921‐927. doi:10.1016/j.athoracsur.2020.09.029 33159862

[10] Chen X , Pang Z , Wang Y , et al. The role of surgery for atypical bronchopulmonary carcinoids tumor: development and validation of a model based on surveillance, epidemiology, and end results (SEER) database. Lung Cancer. 2020;139:94‐102. doi:10.1016/j.lungcan.2019.11.006 31759223

[11] Doll KM , Rademaker A , Sosa JA . Practical guide to surgical data sets: surveillance, epidemiology, and end results (SEER) database. JAMA Surg. 2018;153(6):588‐589. doi:10.1001/jamasurg.2018.0501 29617544

[12] Makuch RW . Adjusted survival curve estimation using covariates. J Chronic Dis. 1982;35(6):437‐443. doi:10.1016/0021-9681(82)90058-3 7042727

[13] Chang IM , Gelman R , Pagano M . Corrected group prognostic curves and summary statistics. J Chronic Dis. 1982;35(8):669‐674. doi:10.1016/0021-9681(82)90019-4 7096530

[14] Mokhayeri Y , Hashemi‐Nazari SS , Khodakarim S , et al. Effects of hypothetical interventions on ischemic stroke using parametric G‐formula. Stroke. 2019;50(11):3286‐3288. doi:10.1161/STROKEAHA.119.025749 31480969

[15] Hu ZH , Peter Gale R , Zhang MJ . Direct adjusted survival and cumulative incidence curves for observational studies. Bone Marrow Transplant. 2020;55(3):538‐543. doi:10.1038/s41409-019-0552-y 31101889 PMC7306148

[16] Kim DH , Shi SM , Carroll D , Najafzadeh M , Wei LJ . Restricted mean survival time versus conventional measures for treatment decision‐making. J Am Geriatr Soc. 2021;69(8):2282‐2289. doi:10.1111/jgs.17195 33901300 PMC8373742

[17] Shi SM , Palmer JA , Newmeyer N , et al. Restricted mean survival time versus conventional effect summary for treatment decision‐making: a mixed‐methods study. J Am Geriatr Soc. 2023;71(2):528‐537. doi:10.1111/jgs.18107 36318788 PMC9957827

[18] Hendifar AE , Marchevsky AM , Tuli R . Neuroendocrine tumours of the lung: current challenges and advances in the diagnosis and management of well‐differentiated disease. J Thorac Oncol. 2017;12(3):425‐436. doi:10.1016/j.jtho.2016.11.2222 27890494

[19] GBD 2019 Risk Factors Collaborators . Global burden of 87 risk factors in 204 countries and territories, 1990–2019: a systematic analysis for the global burden of disease study 2019. Lancet. 2020;396(10258):1223‐1249. doi:10.1016/S0140-6736(20)30752-2 33069327 PMC7566194

[20] Yang Z , Wang Z , Duan Y , Xu S . Clinicopathological characteristics and prognosis of resected cases of carcinoid tumours of the lung. Thoracic Cancer. 2016;7(6):633‐638. doi:10.1111/1759-7714.12377 27755793 PMC5093170

[21] Zhong CX , Yao F , Zhao H , Shi JX , Fan LM . Long‐term outcomes of surgical treatment for pulmonary carcinoids tumours: 20 years' experience with 131 patients. Chin Med J (Engl). 2012;125(17):3022‐3026. 22932173

[22] Dermawan JK , Farver CF . The prognostic significance of the 8th edition TNM staging of pulmonary carcinoids Tumours: a single institution study with long‐term follow‐up. Am J Surg Pathol. 2019;43(9):1291‐1296. doi:10.1097/PAS.0000000000001268 31094922

[23] Wegner RE , Abel S , Hasan S , et al. The role of adjuvant therapy for atypical bronchopulmonary carcinoids. Lung Cancer. 2019;131:90‐94. doi:10.1016/j.lungcan.2019.03.022 31027704

[24] Tsuta K , Raso MG , Kalhor N , Liu DD , Wistuba II , Moran CA . Histologic features of low‐ and intermediate‐grade neuroendocrine carcinoma (typical and atypical carcinoid tumours) of the lung. Lung Cancer. 2011;71(1):34‐41. doi:10.1016/j.lungcan.2010.04.007 20462655

[25] Han B , Sun JM , Ahn JS , Park K , Ahn MJ . Clinical outcomes of atypical carcinoid tumours of the lung and thymus: 7‐year experience of a rare malignancy at single institute. Med Oncol. 2013;30(1):479. doi:10.1007/s12032-013-0479-x 23377986

[26] Caplin ME , Baudin E , Ferolla P , et al. ENETS consensus conference participants. Pulmonary neuroendocrine (carcinoid) tumours: European neuroendocrine tumor society expert consensus and recommendations for best practice for typical and atypical pulmonary carcinoids. Ann Oncol. 2015;26(8):1604‐1620. doi:10.1093/annonc/mdv041 25646366

[27] Yang H , Xiao X , Mei T , Zhou P . Long‐term survival analysis of sublobar resection versus lobectomy for older patients with early‐stage pulmonary carcinoids tumour: a database‐based propensity score‐matched study. Aging Clin Exp Res. 2022;34(8):1925‐1934. doi:10.1007/s40520-022-02112-0 35347580

[28] Glazer ES , Tseng JF , Al‐Refaie W , et al. Long‐term survival after surgical management of neuroendocrine hepatic metastases. HPB (Oxford). 2010;12(6):427‐433. doi:10.1111/j.1477-2574.2010.00198 20662794 PMC3028584

