# Supplementary figures and images for: Prognostic nomogram and epidemiological analysis for lung atypical carcinoid: A SEER database and external validation study
Source: Cancer Med. 2023 Dec 20;13(1):e6794. doi: 10.1002/cam4.6794 (PMC10807636; doi:10.1002/cam4.6794)

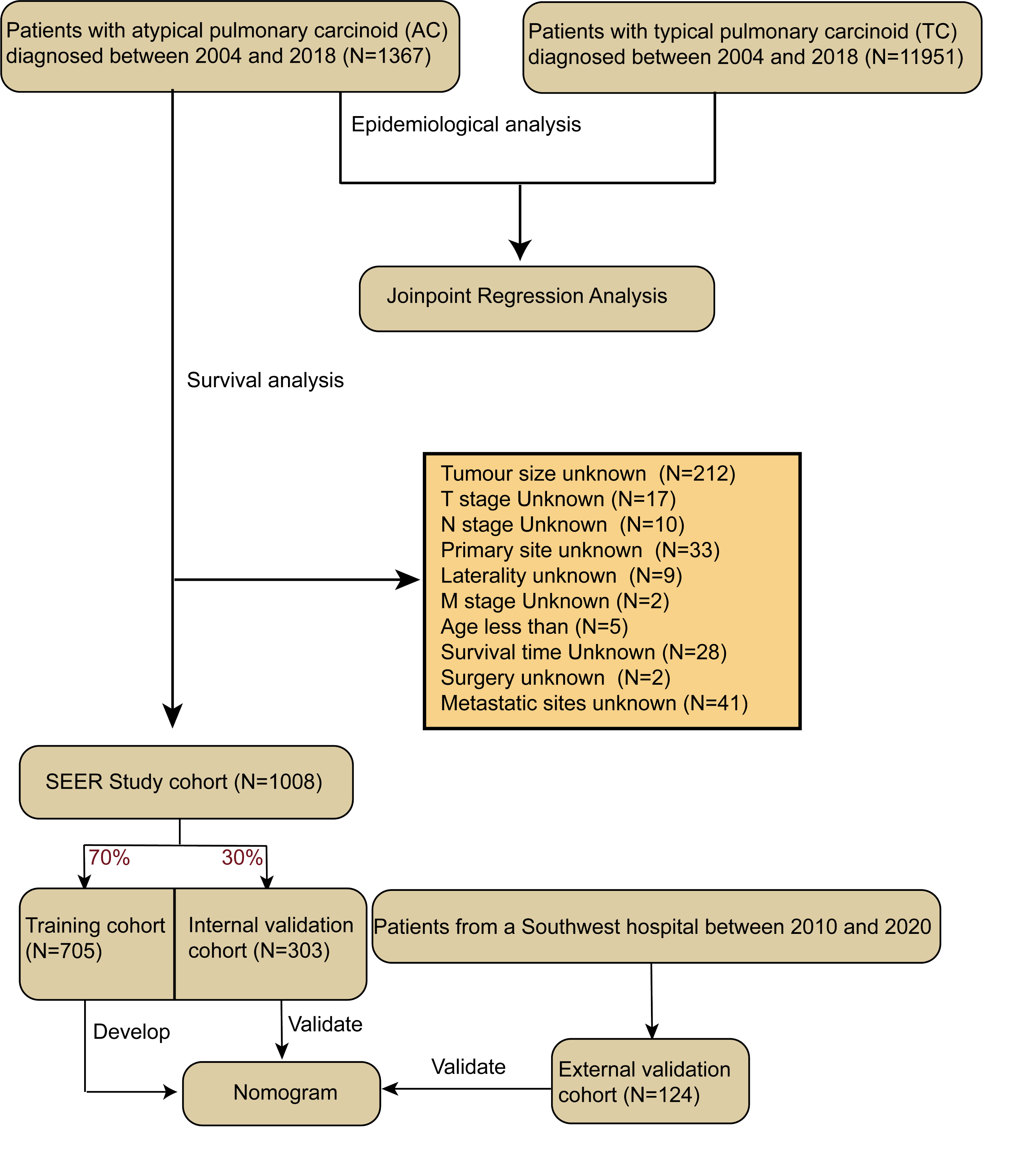

Supplement: Supplementary file 1 — Figure S1. [file CAM4-13-e6794-s002.tif]

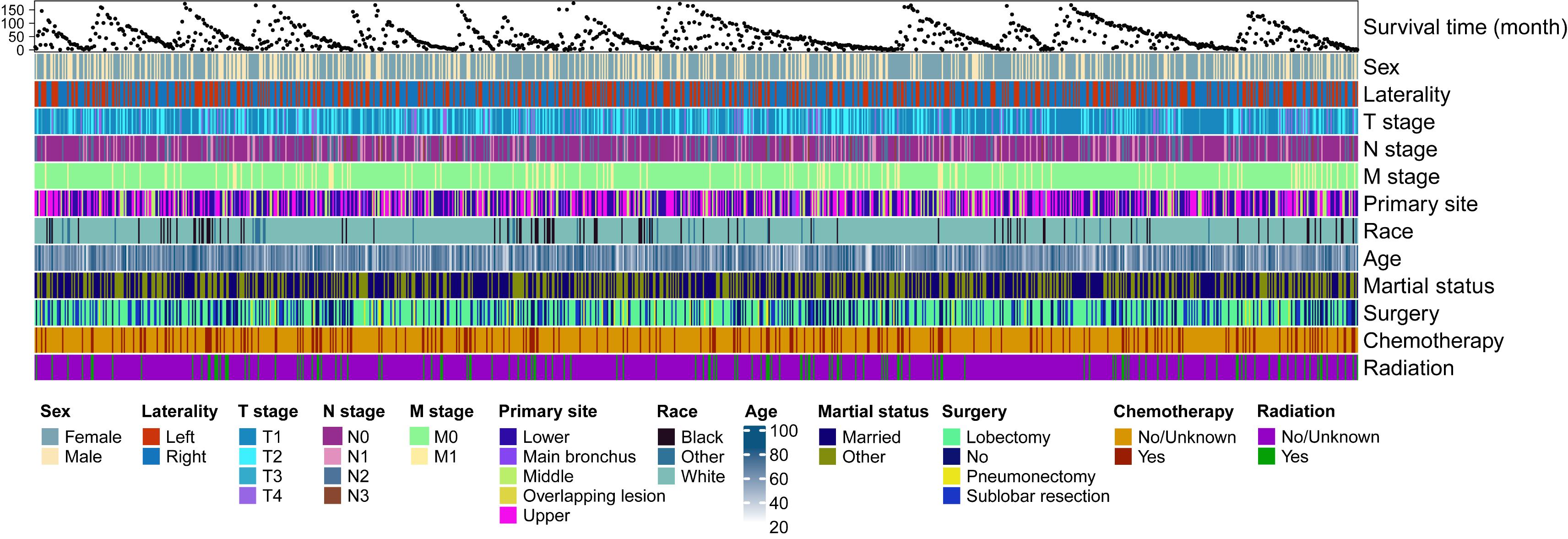

Supplement: Supplementary file 2 — Figure S2. [file CAM4-13-e6794-s004.tif]

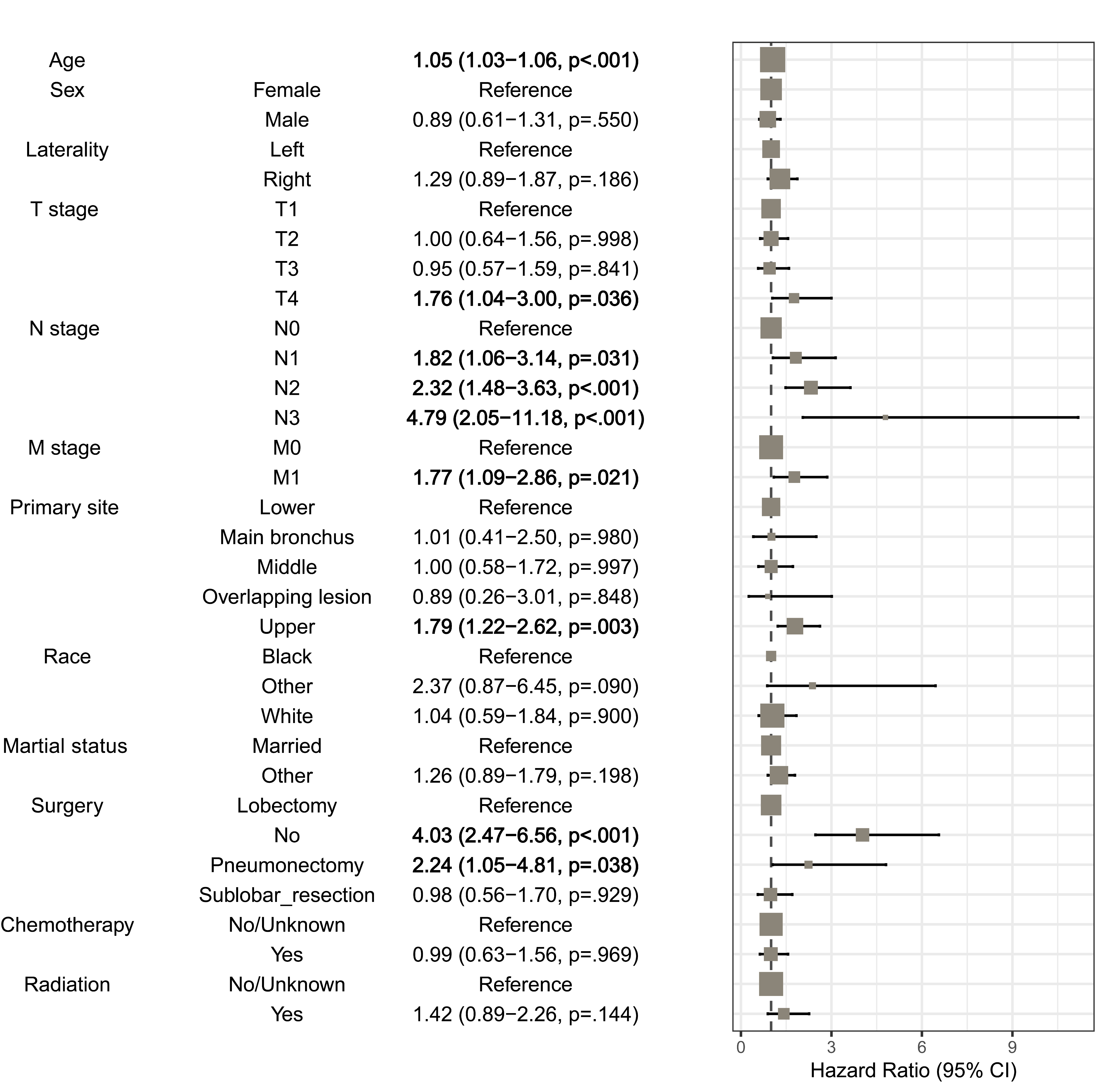

Supplement: Supplementary file 3 — Figure S3. [file CAM4-13-e6794-s005.tif]

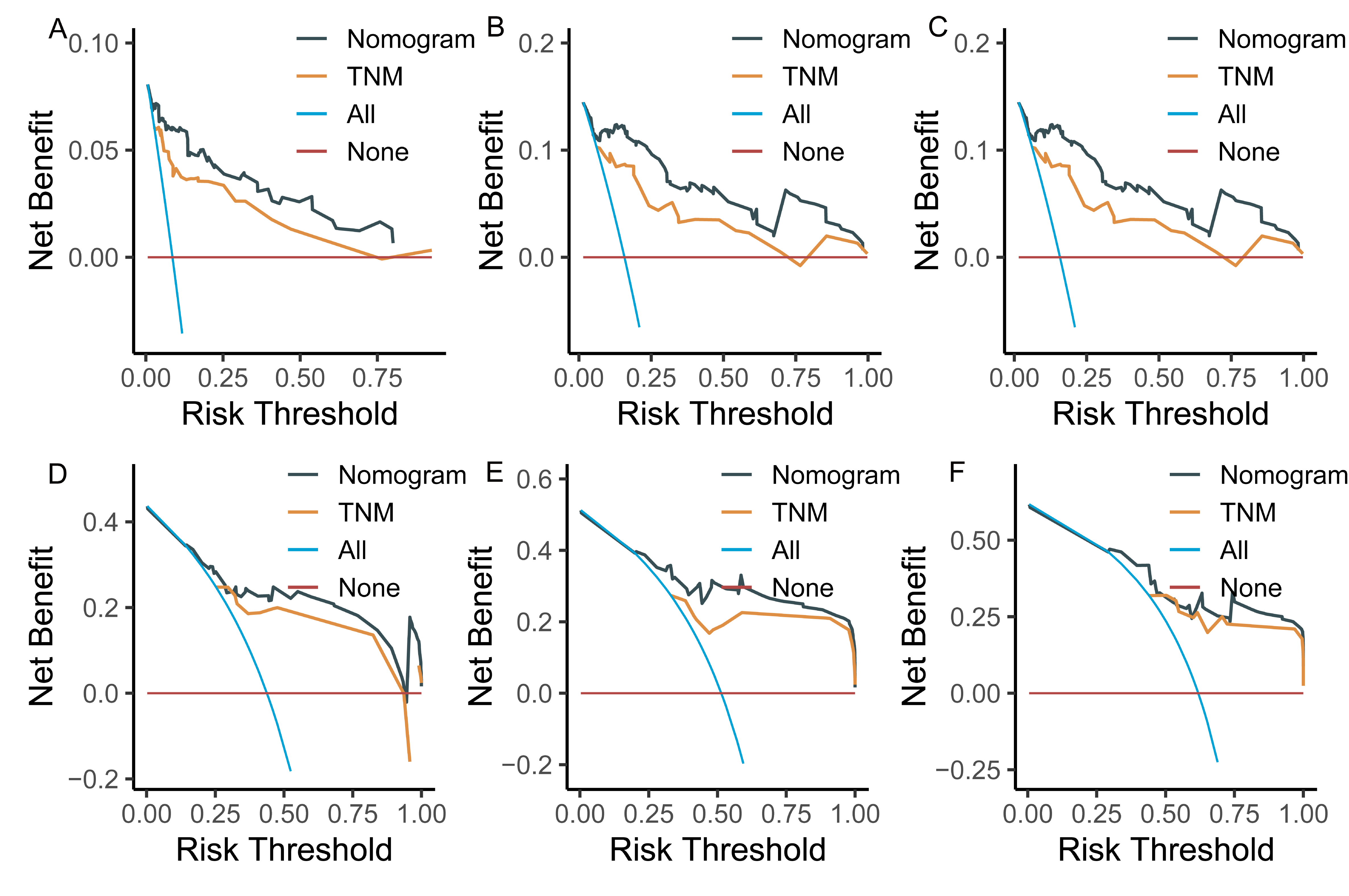

Supplement: Supplementary file 4 — Figure S4. [file CAM4-13-e6794-s006.tif]

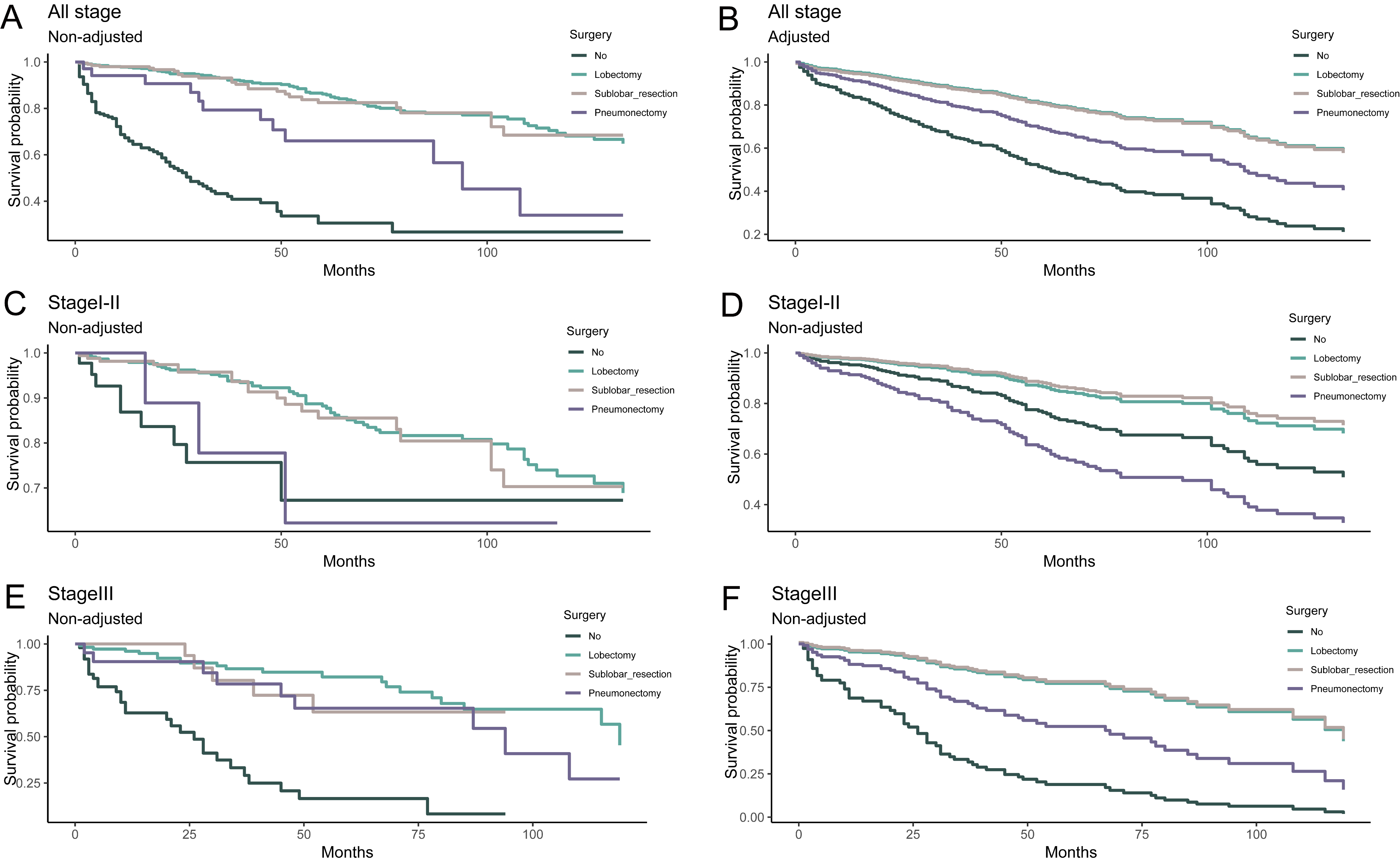

Supplement: Supplementary file 5 — Figure S5. [file CAM4-13-e6794-s001.tif]
