# Supplementary material for: Prognostic nomogram and epidemiological analysis for lung atypical carcinoid: A SEER database and external validation study
Source: Cancer Med. 2023 Dec 20;13(1):e6794. doi: 10.1002/cam4.6794 (PMC10807636; doi:10.1002/cam4.6794)
Supplement: Supplementary file 6 — Table S1. [file CAM4-13-e6794-s007.docx]

**Supplementary Table1**: Association between smoking status and baseline characteristics

| Characteristics | levels | No (N=25) | Yes (N=99) | p value |
| --- | --- | --- | --- | --- |
| T stage | T1 | 11 (44%) | 50 (50.5%) | 0.458 |
|  | T2 | 8 (32%) | 27 (27.3%) |  |
|  | T3 | 5 (20%) | 11 (11.1%) |  |
|  | T4 | 1 (4%) | 11 (11.1%) |  |
| N stage | N0 | 17 (68%) | 63 (63.6%) | 0.94 |
|  | N1 | 4 (16%) | 15 (15.2%) |  |
|  | N2 | 3 (12%) | 17 (17.2%) |  |
|  | N3 | 1 (4%) | 4 (4%) |  |
| M stage | M0 | 21 (84%) | 79 (79.8%) | 0.848 |
|  | M1 | 4 (16%) | 20 (20.2%) |  |
| Sex | Female | 15 (60%) | 25 (25.3%) | 0.002 |
|  | Male | 10 (40%) | 74 (74.7%) |  |
| ACSS | Mean ± SD | 0.4 ± 0.5 | 0.5 ± 0.5 | 0.267 |

ACSS: pulmonary atypical carcinoid specific survival
