# Supplementary material for: Prognostic nomogram and epidemiological analysis for lung atypical carcinoid: A SEER database and external validation study
Source: Cancer Med. 2023 Dec 20;13(1):e6794. doi: 10.1002/cam4.6794 (PMC10807636; doi:10.1002/cam4.6794)
Supplement: Supplementary file 7 — Data S1. [file CAM4-13-e6794-s003.docx]

getpred.DN <-

function (model, newd, set.rms = F)

{

inrange <- T

mclass <- getclass.DN(model)$model.class

if (!mclass %in% c("lm", "glm", "coxph", "ols", "lrm", "Glm",

"cph", "gam", "Gam", "glmnet"))

stop("Unrecognized model object type.")

if (mclass %in% c("ols", "lrm", "Glm", "cph")) {

if (set.rms == T) {

model <- update(model, x = T, y = T, data = data)

}

else {

model <- update(model, x = T, y = T)

}

}

if (mclass %in% c("ols", "lrm", "Glm")) {

m.pred <- predict(model, newdata = newd, se.fit = TRUE)

mpred <- m.pred$linear.predictors

se.pred <- m.pred$se.fit[[1]]

}

if (mclass %in% c("glm", "gam")) {

m.pred <- prediction(model, data = newd, type = "link",

calculate_se = TRUE)

mpred <- m.pred$fit

se.pred <- m.pred$se.fitted

}

if (mclass %in% c("lm", "Gam")) {

m.pred <- prediction(model, data = newd, calculate_se = TRUE)

mpred <- m.pred$fit

se.pred <- m.pred$se.fitted

}

if (mclass %in% c("coxph")) {

if (!any(class(try(prediction(model, data = newd, type = "expected",

calculate_se = TRUE), silent = TRUE)) == "try-error")) {

m.pred <- prediction(model, data = newd, type = "expected",

calculate_se = TRUE)

mpred <- m.pred$fit

se.pred <- m.pred$se.fit

}

else {

inrange = F

mpred <- 0

se.pred <- 0

}

}

if (mclass %in% c("cph")) {

strata.l <- levels(model$strata)

if (length(model$strata) != length(levels(attr(predict(model,

newd, type = "x", expand.na = FALSE), "strata")))) {

levels(model$strata) <- levels(attr(predict(model,

newd, type = "x", expand.na = FALSE), "strata"))

}

m.pred <- suppressWarnings({

survest(model, newdata = newd, times = newd[, all.vars(model$terms)[1]])

})

mpred <- -log(m.pred$surv)

se.pred <- m.pred$std.err

if (mpred == 0) {

inrange = F

}

}

list(pred = mpred, SEpred = se.pred, InRange = inrange)

}

getclass.DN <-

function (model)

{

mfamily <- NA

mclass <- attr(model, "class")[1]

if (mclass == "coxph.null")

stop("Error in model syntax: the model is null")

if (!mclass %in% c("lm", "glm", "coxph", "ols", "lrm", "Glm",

"cph", "gam", "Gam", "glmnet"))

stop("Unrecognized model object type.")

if (mclass %in% c("elnet", "lognet", "multnet", "fishnet",

"coxnet", "mrelnet")) {

mclass <- "glmnet"

mfamily <- attr(model, "class")[1]

}

if (mclass %in% c("glm", "Glm"))

mfamily <- model$family$family

if (mclass == "lrm")

mfamily <- mclass

list(model.class = mclass, model.family = mfamily)

}

ui = bootstrapPage(fluidPage(

titlePanel('Dynamic Nomogram'),

sidebarLayout(sidebarPanel(uiOutput('manySliders'),

checkboxInput('trans', 'Alpha blending (transparency)', value = TRUE),

actionButton('add', 'Predict'),

br(), br(),

helpText('Press Quit to exit the application'),

actionButton('quit', 'Quit')

),

mainPanel(tabsetPanel(id = 'tabs',

tabPanel('Survival plot', plotOutput('plot')),

tabPanel('Predicted Survival', plotlyOutput('plot2')),

tabPanel('Numerical Summary', verbatimTextOutput('data.pred')),

tabPanel('Model Summary', verbatimTextOutput('summary'))

)

)

)))

server = function(input, output){

observe({if (input$quit == 1)

stopApp()})

output$manySliders <- renderUI({

slide.bars <- list()

for (j in 1:length(preds)){

if (preds[[j]]$dataClasses == "factor"){

slide.bars[[j]] <- list(selectInput(names(preds)[j], names(preds)[j], preds[[j]]$v.levels, multiple = FALSE))

}

if (preds[[j]]$dataClasses == "numeric"){

if (covariate == "slider") {

slide.bars[[j]] <- list(sliderInput(names(preds)[j], names(preds)[j],

min = preds[[j]]$v.min, max = preds[[j]]$v.max, value = preds[[j]]$v.mean))

}

if (covariate == "numeric") {

slide.bars[[j]] <- list(numericInput(names(preds)[j], names(preds)[j], value = zapsmall(preds[[j]]$v.mean, digits = 4)))

}}}

if (covariate == "slider") {

slide.bars[[length(preds) + 1]] <-

list(br(), checkboxInput("times", "Predicted Survival at this Follow Up:"),

conditionalPanel(condition = "input.times == true",

sliderInput("tim", tim[1], min = ttim$v.min, max = ttim$v.max, value = ttim$v.mean)))

} else {

slide.bars[[length(preds) + 1]] <-

list(br(), checkboxInput("times", "Predicted Survival at this Follow Up:"),

conditionalPanel(condition = "input.times == true",

numericInput("tim", tim[1], value = zapsmall(ttim$v.mean, digits = 4))))

}

do.call(tagList, slide.bars)

})

a <- 0

old.d <- NULL

new.d <- reactive({

input$add

input.v <- vector("list", length(preds) + 1)

input.v[[1]] <- isolate({ input[["tim"]] })

names(input.v)[1] <- tim[1]

for (i in 1:length(preds)) {

input.v[[i+1]] <- isolate({

input[[names(preds)[i]]]

})

names(input.v)[i+1] <- names(preds)[i]

}

out <- data.frame(lapply(input.v, cbind))

if (a == 0) {

wher <- match(names(out), names(input.data))

out <- out[wher]

input.data <<- rbind(input.data, out)

}

if (a > 0) {

wher <- match(names(out), names(input.data))

out <- out[wher]

if (!isTRUE(compare(old.d, out))) {

input.data <<- rbind(input.data, out)

}}

a <<- a + 1

out

})

p1 <- NULL

old.d <- NULL

data2 <- reactive({

if (input$add == 0)

return(NULL)

if (input$add > 0) {

if (!isTRUE(compare(old.d, new.d()))) {

OUT <- isolate({

new.d <- cbind(st.ind = 1, new.d())

names(new.d)[1] <- tim[2]

DNpred <- getpred.DN(model, new.d)

mpred <- DNpred$pred

se.pred <- DNpred$SEpred

pred <- mlinkF(mpred)

if (is.na(se.pred)) {

lwb <- NULL

upb <- NULL

} else {

lwb <- sort(mlinkF(mpred + cbind(1, -1) * (qnorm(1 - (1 - clevel)/2) * se.pred)))[1]

upb <- sort(mlinkF(mpred + cbind(1, -1) * (qnorm(1 - (1 - clevel)/2) * se.pred)))[2]

if (upb > 1) {

upb <- 1

}}

if (ptype == "st") {

d.p <- data.frame(Prediction = zapsmall(pred, digits = 2),

Lower.bound = zapsmall(lwb, digits = 2),

Upper.bound = zapsmall(upb, digits = 2))

}

if (ptype == "1-st") {

d.p <- data.frame(Prediction = zapsmall(1-pred, digits = 2),

Lower.bound = zapsmall(1-upb, digits = 2),

Upper.bound = zapsmall(1-lwb, digits = 2))

}

old.d <<- new.d[,-1]

data.p <- cbind(d.p, counter = TRUE)

if (DNpred$InRange){

p1 <<- rbind(p1[,-5], data.p)

} else{

p1 <<- rbind(p1[,-5], data.frame(Prediction = NA, Lower.bound = NA, Upper.bound = NA, counter = FALSE))

}

p1

})

} else {

p1$count <- seq(1, dim(p1)[1])

}}

p1

})

s.fr <- NULL

old.d2 <- NULL

b <- 1

dat.p <- reactive({

if (isTRUE(compare(old.d2, new.d())) == FALSE) {

try.survfit <- !any(class(try(survfit(model, newdata = new.d()), silent = TRUE)) == "try-error")

if (try.survfit){

fit1 <- survfit(model, newdata = new.d())

}

if (n.strata == 0) {

sff <- data.frame(summary(fit1)[c("time", "n.risk", "surv")])

sff <- cbind(sff, event=1-sff$surv, part = b)

if (sff$time[1] != 0){

sff <- rbind(data.frame(time=0, n.risk=sff$n.risk[1] ,surv=1, event=0, part=sff$part[1]), sff)

}}

if (n.strata > 0) {

nam <- NULL

new.sub <- T

for (i in 1:(dim.terms-1)) {

if (preds[[i]]$dataClasses == "factor"){

if (preds[[i]]$IFstrata){

nam0=paste(new.d()[[names(preds[i])]], sep = '')

if (new.sub) {

nam <- paste(nam0)

new.sub <- F

} else {

nam <- paste(nam, ', ', nam0, sep = '')

}}}}

if (try.survfit){

sub.fit1 <- subset(as.data.frame(summary(fit1)[c("time", "n.risk", "strata", "surv")]), strata == nam)

} else{

sub.fit1 <- data.frame(time=NA, n.risk=NA, strata=NA, surv=NA, event=NA, part=NA)[0,]

}

if (!try.survfit){

message("The strata levels not found in the original")

sff <- cbind(sub.fit1, event=NULL, part = NULL)

b <<- b - 1

} else{

sff <- cbind(sub.fit1, event=1-sub.fit1$surv, part = b)

if (sff$time[1] != 0) {

sff <- rbind(data.frame(time=0, n.risk=sff$n.risk[1], strata=sff$strata[1] ,surv=1, event=0, part=sff$part[1]), sff)

}

sff$n.risk <- sff$n.risk/sff$n.risk[1]

}

sff$n.risk <- sff$n.risk/sff$n.risk[1]

}

s.fr <<- rbind(s.fr, sff)

old.d2 <<- new.d()

b <<- b + 1

}

s.fr

})

dat.f <- reactive({

if (nrow(data2() > 0))

cbind(input.data, data2()[1:3])

})

# KM plot

output$plot <- renderPlot({

data2()

if (input$add == 0)

return(NULL)

if (input$add > 0) {

if (ptype == "st") {

if (input$trans == TRUE) {

pl <- ggplot(data = dat.p()) +

geom_step(aes(x = time, y = surv, alpha = n.risk, group = part), color = coll[dat.p()$part])

}

if (input$trans == FALSE) {

pl <- ggplot(data = dat.p()) +

geom_step(aes(x = time, y = surv, group = part), color = coll[dat.p()$part])

}}

if (ptype == "1-st") {

if (input$trans == TRUE) {

pl <- ggplot(data = dat.p()) +

geom_step(aes(x = time, y = event, alpha = n.risk, group = part), color = coll[dat.p()$part])

}

if (input$trans == FALSE) {

pl <- ggplot(data = dat.p()) +

geom_step(aes(x = time, y = event, group = part), color = coll[dat.p()$part])

}}

pl <- pl + ylim(0, 1) + xlim(0, max(dat.p()$time) * 1.05) +

labs(title = "Estimated Survival Probability", x = "Follow Up Time", y = "S(t)") + theme_bw() +

theme(text = element_text(face = "bold", size = 10), legend.position = "none", plot.title = element_text(hjust = .5))

}

print(pl)

})

output$plot2 <- renderPlotly({

if (input$add == 0)

return(NULL)

if (is.null(new.d()))

return(NULL)

lim <- c(0, 1)

yli <- c(0 - 0.5, 10 + 0.5)

input.data = input.data[data2()$counter,]

in.d <- data.frame(input.data)

xx=matrix(paste(names(in.d), ": ",t(in.d), sep=""), ncol=dim(in.d)[1])

text.cov=apply(xx,2,paste,collapse="<br />")

if (dim(input.data)[1] > 11)

yli <- c(dim(input.data)[1] - 11.5, dim(input.data)[1] - 0.5)

dat2 <- data2()[data2()$counter,]

dat2$count = seq(1, nrow(dat2))

p <- ggplot(data = dat2, aes(x = Prediction, y = count - 1, text = text.cov,

label = Prediction, label2 = Lower.bound, label3=Upper.bound)) +

geom_point(size = 2, colour = coll[dat2$count], shape = 15) +

ylim(yli[1], yli[2]) + coord_cartesian(xlim = lim) +

labs(title = "95% Confidence Interval for Response",

x = "Survival probability", y = "") + theme_bw() +

theme(axis.text.y = element_blank(), text = element_text(face = "bold", size = 10))

if (is.numeric(dat2$Upper.bound)){

p <- p + geom_errorbarh(xmax = dat2$Upper.bound, xmin = dat2$Lower.bound,

size = 3, height = 0.4, colour = coll[dat2$count])

} else{

message("Confidence interval is not available as there is no standard errors available by 'coxph' ")

}

if (ptype == "st") {

p <- p + labs(title = paste(clevel * 100, "% ", "Confidence Interval for Survival Probability", sep = ""),

x = DNxlab, y = DNylab)

}

if (ptype == "1-st") {

p <- p + labs(title = paste(clevel * 100, "% ", "Confidence Interval for F(t)", sep = ""),

x = DNxlab, y = DNylab)

}

gp=ggplotly(p, tooltip = c("text","label","label2","label3"))

gp$elementId <- NULL

dat.p()

gp

})

output$data.pred <- renderPrint({

if (input$add > 0) {

if (nrow(data2() > 0)) {

stargazer(dat.f(), summary = FALSE, type = "text")

}}

})

output$summary <- renderPrint({

summary(model)

})

}
